# A novel mobile application to determine mandibular and tongue laterality discrimination in women with chronic temporomandibular disorder

**DOI:** 10.4317/medoral.23785

**Published:** 2020-07-23

**Authors:** Marta Carlota Diaz-Saez, Alfonso Gil-Martínez, Irene Iglesias González, Jesús Kim Lee, José Luis del-Castillo Pardo de-Vera, José Luis Cebrián Carretero, Hector Beltran-Alacreu

**Affiliations:** 1 Physiotherapy Department, Centro Superior de Estudios Universitarios La Salle, Universidad Autónoma de Madrid, Spain; 2 CranioSPain Research Group, Centro Superior de Estudios Universitarios La Salle, Universidad Autónoma de Madrid, Spain; 3 Unit of physiotherapy, Hospital La Paz Institute for Health Research (IdiPaz), Madrid, Spain; 4 Maxilofacial and Oral Surgery Department, Hospital Universitario La Paz, Madrid, Spain

## Abstract

**Background:**

Chronic pain from temporomandibular disorders (TMDs) is caused by a somatosensory disturbance due to sustained activation of central nervous system nociceptive pathways, which can induce changes in neuroplasticity in the thalamus, basal ganglia and limbic system, as well as disturbances in the somatosensory, prefrontal and orbitofrontal cortex and cognitive impairment. The main objective of this study was to determine the discrimination capacity of mandibular and tongue laterality between women with chronic TMDs and asymptomatic women.

**Material and Methods:**

This descriptive-comparative study examined 2 groups with a total of 30 women. All participants were between the ages of 23 and 66 years and were assigned to the chronic TMD group or the asymptomatic group according to the inclusion criteria. We employed a mobile application developed specifically for this study to measure the accuracy and reaction time (RT) of mandibular and tongue laterality discrimination.

**Results:**

The chronic TMD group had a lower success rate in laterality discrimination (mean mandibular accuracy of 40% and mean tongue accuracy of 67%) than the asymptomatic group (mean mandibular accuracy of 61% and mean tongue accuracy of 90%). These results showed statistically significant differences between the groups for mandibular laterality discrimination (d, 1.14; *p*<0.01) and tongue laterality discrimination (d, 0.79; *p*=0.03). The asymptomatic group had faster RTs than the chronic TMD group. The data revealed statistically significant differences for the right mandibular RT (d, 0.89; *p*=0.02) and right tongue RT (d, 0.83; *p*=0.03). However, there were no significant differences for left mandibular and left tongue RT.

**Conclusions:**

We found that the women with chronic TMDs had a lower success rate and slower RTs in the discrimination of mandibular laterality when compared with the asymptomatic women.

** Key words:**Mobile application, tongue, chronic temporomandibular disorder, pain, lateral discrimination, cortical representation, reaction time.

## Introduction

Temporomandibular disorders (TMDs) are a subset of craniofacial disorders and are defined as disturbances that affect masticatory muscles, temporomandibular joints (TMJ) and head/neck musculoskeletal structures (1,2). TMDs can generate symptoms such as palpation sensitivity, limited or asymmetric range of motion and TMJ noise (3). Patients with TMDs report symptoms ranging from earache, tinnitus, bruxism, dizziness and open-mouth difficulty to neck pain and headaches (3). TMJ pain is relatively common, affecting approximately 3%–15% of the general population (4) and 7% of adolescents (5) and becoming chronic for 1.6% of the population. TMJ pain is more prevalent in women (15% of TMD cases) than in men (8% of cases). A number of studies have observed a strong correlation between chronic TMD and psychosocial factors, resulting in a negative impact on the patients’ quality of life (6).

Chronic pain from TMDs is caused by a somatosensory disturbance due to sustained activation of central nervous system nociceptive pathways. The pain can also start via various inputs to cervical craniofacial tissues at the periphery (7). The phenomenon of chronicity can induce neuroplastic changes in the thalamus, basal ganglia and limbic system, as well as disturbances in the somatosensory cortex, prefrontal cortex and orbitofrontal cortex, resulting in cognitive impairment (8). Chronic craniofacial pain can therefore result in self-face perception distortion, contributing to the chronification process and maintaining pain over time (9). In addition to affecting other parts of the body (10–13), this chronic pain can affect cerebral areas related to body image perception, such as the primary and secondary somatosensory cortexes and the primary motor cortex, as demonstrated by various studies (14). The chronic pain can result in disability and disturbances in function, pain perception, accuracy and reaction time (RT) in laterality discrimination (LD) (15). To our knowledge, however, there have been no studies to date that have evaluated these variables in patients with chronic TMDs.

Various research studies have estimated that the socioeconomic burden of TMD reaches over 4 billion USD per year (16). Early assessment and intervention could therefore considerably reduce these costs. Moreover, therapies based on the association between cortical reorganization and pain and corporal image distortion appear to be effective for modulating pain (14). León-Hernández *et al*. developed an LD training program for patients with non-specific chronic neck pain, achieving improved cervical range of motion and joint position sense after performing the LD task (17). Using positron emission tomography, Parson *et al*. observed that participants solved visual shape tasks by activating the frontal, parietal and cerebellar regions but not the primary somatosensory cortex and motor cortex. These findings lead to the conclusion that mental imagery is not limited to the perceptual system of the stimuli that appears in the task (18). Mosley conducted studies with image discrimination tasks to train study participants in the LD of various body areas, providing a new tool for treating patients to reduce pain and disability resulting from complex pain syndromes (10). Further research in this field is needed to clarify the factors involved in this condition and to find new approach models.

The aim of this research study was to determine the ability to discriminate mandibular and tongue laterality in women with chronic TMDs compared with asymptomatic women. We also sought to determine the RT for mandibular LD (MLD) and tongue LD (TLD) between patients with chronic TMDs and asymptomatic participants.

## Material and Methods

We conducted a descriptive-comparative study based on the 2009 Strengthening the Reporting of Observational studies in Epidemiology Statement (19). The ethics committee of La Paz University Hospital (HULP) approved this research study (project code PI-3077). The patients with TMDs were recruited from the Oral and Maxillofacial Surgery Service of the TMJ Department, while the asymptomatic participants were recruited from the patients’ relatives and companions or from HULP staff.

The research team consisted of an experienced (more than 10 years) maxillofacial surgeon from the HULP Temporomandibular Joint Unit, 2 information technology (IT) engineers, 3 experienced physical therapists and 3 assessors. The ad hoc mobile application, which evaluated MLD and TLD and RT, was developed by the IT engineers in collaboration with the clinicians, from the “Cognitive” prototype to the final 3.1 version. The maxillofacial surgeon selected the patients according to the inclusion and exclusion criteria. The 3 assessors were trained to conduct the entire procedure by an experienced physical therapist.

- Selection criteria and participant description

We recruited 30 participants from April 2019 to October 2019. The women with chronic TMDs were included based on the following criteria: 23–66 years of age, female, previously diagnosed by the maxillofacial surgeon of the HULP Temporomandibular Joint Unit, diagnosis based on the Diagnostic Criteria for Temporomandibular Disorders (DC/TMD) (20) and disease chronicity (>6 months).

Participants were recruited to the asymptomatic group if they m*et al*l of the following criteria: 23–66 years of age, female, no history of craniofacial/temporomandibular/neck pain, no facial palsy caused by a primary muscle disorder and no significant history of chronic pain disorder.

The medically related exclusion criteria were the same for both groups: undergoing physical therapy for the neck or craniofacial region, surgery or a history of trauma to the neck/head/face/tongue/teeth/jaw, cancer or active infection of the neck/head/mouth, rheumatic disorders, neurological disorders and pregnancy. Additional exclusion criteria were the inability to use the mobile application, limited accessibility or usability of the application or mobile device, inability to understand the inform consent document, inability to understand the study procedure and inability to understand the language.

All medication was allowed as concomitant treatment, except for drugs that affected cognition or reaction capacity (psychotropics and narcotic drugs), which were considered an exclusion criterion. Participants were not allowed to undergo physical therapy treatment during the implementation of this study.

- Study description

After the screening, each participant signed an inform consent document and was assigned to the either the TMD group or the asymptomatic group (AG), as appropriate. The following procedure was applied to both groups:

The participants were measured in a single session divided in two separate parts. Before starting the test, the psychosocial and somatosensory variables (such as pain and orofacial disability) and the functional state of the jaw were measured using the craniofacial pain and disability inventory (CF-PDI). The first part consisted of measuring the right and left MLD using the “Cognitive” application (which is based on Recognise™; www.noigroup.com, Adelaide, Australia). The laterality discrimination is the ability to determine if our brain is able to know what side of our body we are seen when we face a person. In this phenomenon, mental rotation is required too. This could lead us to know if there is a corporal image distortion or not. For that purpose, the “Cognitive” application mixed fifty images of right and left mandibular lateral deviations appeared in the interface and the participants had to select if the image showed a right mandibular lateral deviation or a left mandibular lateral deviation before 5 seconds. The participants had a button with the word left and right. After the last image, the interface showed the number of successes, failures, nulls and the reaction time mean. The participants then rested for 5 minutes. The second part consisted of measuring the right and left TLD, once again using the “Cognitive” application which showed the same as before but with images of right and left tongue lateral deviations.

- Outcome measurements

**Figure 1:**
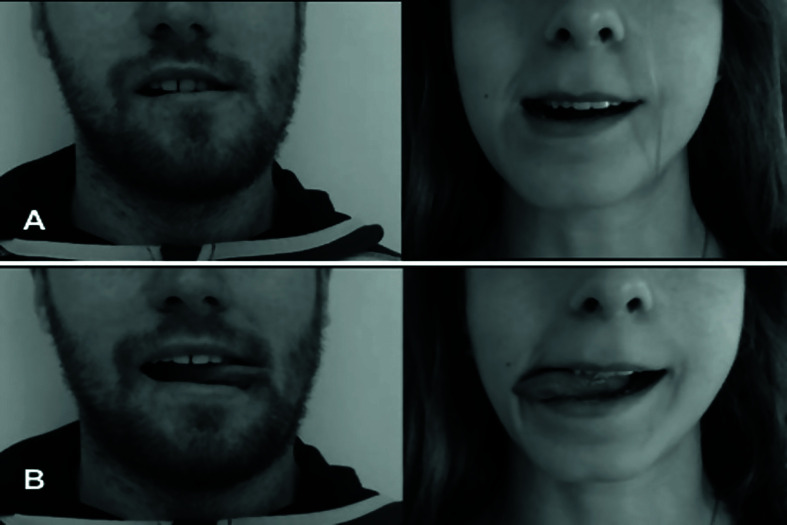
A, left/right mandibular laterality discrimination; B, left/right tongue laterality discrimination.

At the start of the study, we collected the participants’ sociodemographic data (age, sex, standard medications and educational level). The chronic TMD group was asked about the TMD component and the disease’s chronicity. The primary endpoints were mandibular and TLD and reaction time, all of which were measured using the “Cognitive” mobile application. The application is intended for non-commercial use only and contains several images for left/right mandibular and tongue deviations/laterality (Fig. 1). The application allows some of the parameters to be changed, with the option of choosing time between images, only mandibular images, only tongue images or both simultaneously. The total number of images to be shown could be changed, as could be the time limit for displaying the images and the percentage rotation of these images. In the future, these parameters could be changed by the therapist or by the patient. This is could be really useful for the therapist to have an assessment of the subject and control the progression and the difficulty of the treatment. Likewise, this could allow patients to train with the application at home under the recommendations of their therapist. The application records the participants’ success and failure rates after they had pressed the button that appears when the images are displayed.

The reaction time which is the time since the image appears in the screen until the participant select the button left or right, was recorded in seconds.

The secondary endpoints were craniofacial pain and disability, which were measured using the CF-PDI, an inventory that consists of 21 items with a total score ranging from 0 to 63 points based on 2 factors. The inventory measures pain and disability, assesses the functional state of the jaw, has shown good reliability and is the only such inventory validated for the Spanish populations (21).

- Procedure

At the start of the study, each participant was informed of the inclusion and exclusion criteria and given an informed consent form explaining the study procedure. The participants then filled out their epidemiologic and TMJ data (age, sex, educational level, medication, TMD component, duration of symptoms and craniofacial disability) on a data collection sheet, which was then transferred to an Excel sheet accessible only by the assessors. The patients with chronic TMD were then provided with a CF-PDI to measure orofacial pain, disability and jaw function. The participants were then divided into their respective groups. Each participant took a seat and began the intervention using the smartphone-based “Cognitive” application. The procedure was divided into two parts: MLD and TLD. In each part, the assessor entered the parameters into the application for the selection of the limit time and the type of images. The first part consisted of selecting images doing right or left mandibular lateral deviation among 50 randomly mixed mandibular images, with a 5-second time limit to select the correct answer. If the participant exceeded the time limit, the image changed to another and the application considered the answer as null. The participant’s RT was recorded along with the number of successes and failures. The participants were then given a 5-min rest before starting the next test to prevent them from learning the process. During the second part of the study, the participants performed the same procedure as in the first part but for right and left tongue lateral deviations, with the main difference being that the application presented 50 randomly mixed images of right and left tongue lateral deviations.

- Sample size calculation

We estimated the sample size using G*Power 3.1.7 (University of Dusseldorf, Germany) for Windows (22) and considered the sample size calculation as a power calculation to detect intergroup differences in the primary endpoints for 2 independent groups. We estimated 2 groups with 15 participants per group to achieve 80% statistical power (1-â error probability) with an á error probability of 0.05. We calculated a Cohen d effect size of 0.94 in a previous pilot study based on the primary endpoint (mandibular discrimination) between the groups.

- Statistical analysis

To perform the statistical analysis, we employed the Statistical Package for Social Sciences (SPSS 20, SPSS Inc., Chicago, IL, USA). We employed the Shapiro-Wilk test to evaluate the normal distribution of the variables for samples with fewer than 50 participants and employed descriptive statistics to summarize the data for continuous variables, which are presented as mean ± standard deviation (SD) with 95% confidence intervals (CI). The categorical variables are presented as absolute (number) and relative frequencies (percentage). With a 95% confidence interval, we considered values with *p*<0.05 as statistically significant. We employed Fisher’s exact test (2x2 Table) and the chi-squared test with residual analysis to compare the qualitative variables. To compare the quantitative variables for independent samples, we employed Student’s t-test. We calculated effect sizes (d) according to Cohen´s method, in which the magnitude of the effect was classified as small (0.20 to 0.49), medium (0.50 to 0.79) or large (0.8) (Cohen, 1988). We employed the Pearson correlation coefficient for the correlation analysis, classifying the values as small (r=0.10), moderate (r=0.30), moderately large (r=0.50) and large (r=0.70).

## Results

Thirty women met the inclusion criteria for participating in the study and were assigned to one of two groups: chronic TMD group and AG. The mean age (± standard deviation [SD]) of the chronic TMD group was 45.07±13.03 years (range, 23–65 years), and the most frequent educational level was having completed high school (5 patients, 33.3%). The most frequent TMD component was the mixed component (7 patients, 46.7%). Four patients (26.7%) were taking medications during the intervention. The mean TMD chronicity was 95.69±94.50 months.

The mean age of the AG was 46.4±12.84 years (range, 30–66 years), and the most frequent educational level was having completed university (8 participants, 53.3%) followed by completing high school (5 participants, 33.3%).

There were no statistically significant differences (*p*>0.05) between the two groups in terms of the previously mentioned descriptive variables.

- Mandibular and tongue laterality discrimination

The chronic TMD group had a lower success rate for LD (mean MLD, 40%; mean TLD, 67%) than the AG (mean MLD, 61%; mean TLD, 90%). The results revealed significant differences between the groups for MLD (d, 1.14; *p*<0.01) and TLD (d, 0.79; *p*=0.03) (Table 1). There were significant differences for all variables.

- Reaction time for mandibular and tongue laterality discrimination

The AG was faster than the chronic TMD group in terms of mandibular and tongue RT. There were significant differences for the right mandibular RT (d, 0.89; *p*=0.02) and for the right tongue RT (d, 0.83; *p*=0.03). However, there were no significant differences for the left mandibular and left tongue RT (*p*>0.05). There were no statistically significant differences between the groups for the total times (successes and failures) in either the mandible or tongue. Table 2 presents a breakdown of RTs for the right and left mandible and the right and left tongue. The chronic TMD group and the AG spent more time selecting the mandibular laterality images than the tongue laterality images.

**Table 1:**
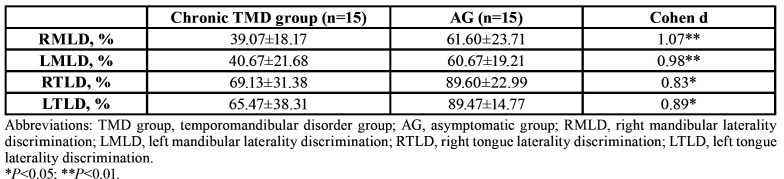
Mandibular and Tongue Laterality Discrimination Rates.

**Table 2:**
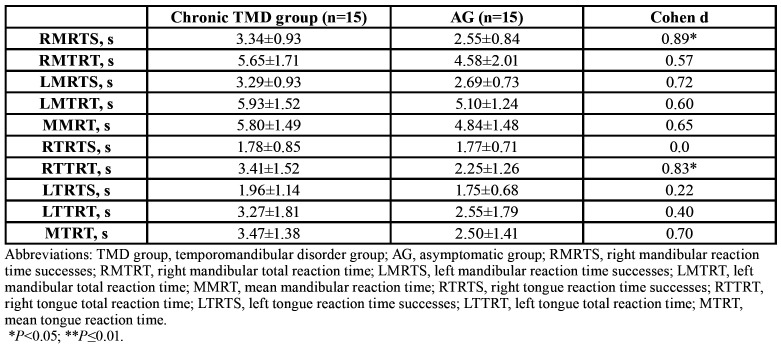
Mandibular and Tongue Reaction Times (in seconds).

- Correlations

The Pearson correlation coefficient indicated a relationship between a number of the study variables (Table 3). There were strong positive correlations between mandibular RT and tongue RT for the AG (r=0.83; *p*<0.01) but not for the chronic TMD group. The study observed a strong positive correlation between right TLD and left TLD for both groups (r=0.89 and r=0.75 for the TMD group and AG, respectively; *p*<0.01 for both). For the chronic TMD group and AG, the analysis found strong negative correlations between TLD and tongue RT (r=-0.70 [p<0.01] and r=-0.63 [p=0.01], respectively). MLD was positively correlated with TLD (r=-0.68; *p*=0.01) for the chronic TMD group but not for the AG. There were strong correlations between age and RT for both groups, and age was positively correlated to mandibular and tongue RT (r=0.63 [p=0.01] and r=0.80 [p<0.01], respectively). The chronic TMD group also presented moderate negative correlations for MLD and mandibular and tongue RT (r=-0.59 and r=-0.58 [p=0.02 for both], respectively). There were no significant correlations (*p*>0.05) between the primary endpoints (MLD, TLD and RT) and the secondary endpoint (CF-PDI) except for RT. The analysis found a strong positive correlation for tongue RT (r=0.52; *p*=0.05) and for right mandibular RT (r=0.55; *p*=0.03) with CF-PDI in the chronic TMD group.

**Table 3:**
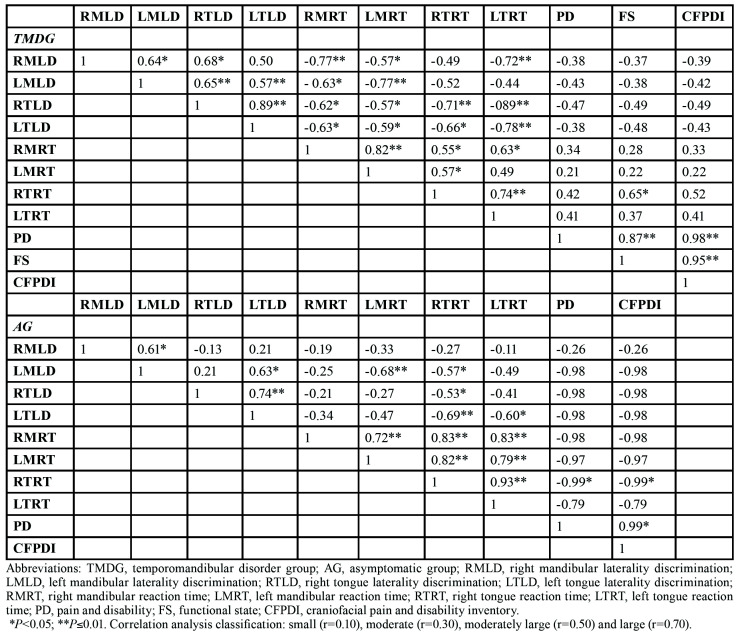
Correlations.

## Discussion

The main objective of this research study was to compare the MLD and TLD capacity between women with chronic TMD and asymptomatic women. The study found significant differences between the chronic TMD group and AG for MLD, TLD and RT, except for left mandibular and tongue RT. These results are scientifically relevant, given the scarce available evidence that has compared these endpoints between patients with chronic TMD and asymptomatic individuals. Von Piekartz *et al*. concluded that patients with facial pain perform more poorly in right and left facial tasks, due to the interruption of cortical motor processing of facial tasks in such patients (23). The authors hypothesized that this conclusion could also apply to patients with TMD. Our results agree with this hypothesis, given that we found lower accuracy in the chronic TMD group for MLD and TLD compared with the AG. We also found that higher pain and disability was related to slower mandibular and tongue RT.

Both groups had slower RTs for right and left MLD than for right and left TLD, findings similar to those reported by Roux *et al*. who concluded that the cortical representation of the tongue occupies more space in the cortex than that of the jaw and nearing the space taken up with the representation of the hand, although the hand has a different representation (24). This finding could explain the greater accuracy and faster RT for the tongue than for the jaw. There is evidence indicating disturbances in the right and left cortical representation of the face and head in TMD and craniofacial pain, as well as chronic low back pain, chronic cervical pain, chronic head pain and chronic leg or foot pain following amputations (23). Our study showed a disorder in the right and left MLD and TLD in women with chronic TMDs, leading to the hypothesis that diseases with chronic pain could provoke distorted body images at the cortical level (25), which could be explained by an increase in sensitization in the region and disinhibition due to the pain, thus altering the recognition of various parts of the body related to the painful region. Von Piekartz *et al*. demonstrated that chronic pain affects the cortical representation of the painful area, in this case, the face (23).

Our chronic TMD group had longer mandibular and tongue RT, with significant differences between right mandibular and tongue RTs but not between left mandibular and tongue RTs. Compared with other studies, our study demonstrated longer RTs for patients with chronic pain than for asymptomatic participants, indicating that patients with chronic pain take longer to react than asymptomatic subjects. The absence of significant differences for left mandibular and tongue RTs could be due to the right-side dominance and to the location of the pain (25).

Our results found strong positive correlations between mandibular and tongue RTs for AG but not for the chronic TMD group, leading to the hypothesis that the TMD component could explain the lack of correlation in mandibular and tongue RTs. Schiffman *et al*. confirmed that TMDs are a heterogenous group that are frequently associated with one or more persistent pain conditions that affect patients differently (20). Our study also found moderate correlations between age and RTs, findings similar to those reported by other studies that have correlated age with overall RT, as well as lower accuracy with age (25,26). Studies have also shown that older individuals take longer to perform simple reaction tasks and have slower RTs. It is important to highlight that an individual’s RT has an inherent right and left discrimination component. Complex tasks require greater motor planning, motor structuring and motor execution and worsen with age (13). Our study revealed a negative correlation between LD and RT. The chronic TMD group presented poorer LD and longer RTs than the AG, results that agree with other studies that found a relationship between general movement accuracy and RT. Pelletier *et al*. observed that patients with wrist and hand pain had longer RTs, which were related to lower LD (27).

Lastly, we found positive correlations between right TLD and left TLD. The study by Roux *et al*. suggests that cortical jaw representation has greater incidence on the right side than the left, occupying a larger area of the cortex. However, the authors’ results show that cortical tongue representation is almost equivalent in the two brain hemispheres (24). These findings match our positive correlation between right TLD and left TLD.

In conclusion, women with chronic TMDs have a lower success rate in MLD and TLD, with MLD inferior to TLD. Moreover, the chronic TMD group had longer LD RTs.

- Clinical implications

The aim of the study was to demonstrate that LD and RTs for patients with chronic TMDs are affected by chronic pain. Based on this objective, we have begun future research lines with the objective of preventing motor function disorders of the jaw and tongue. We also seek to develop an advanced version of the application employed in this study, which would enable a better diagnosis for patients with chronic TMDs and offer a specific treatment method related to the TMD disturbances. Future research should employ the application as a treatment tool combined with other techniques such as graded motor imagery, which could provide interesting avenues for various research groups in this field.

We also need to consider the patients’ psychosocial factors as measurable outcomes due to the chronicity and likelihood of disturbances in LD and RTs in patients with chronic TMDs. Finally, the correlation between MLD and TLD in patients with TMDs could be of benefit in dealing with jaw movement disorders, given that the jaw and tongue are two separate structures; thus, if the jaw presents pain or disorders, the tongue may be used for certain treatments to help improve these altered jaw movements.

- Limitations

The present study has various limitations, the first of which is related to the importance of medication in this study, given that a number of analgesic drugs (psychotropics and narcotics) can affect the patients’ cognitive level, RTs and other TMD variables (such as progression, chronicity and location). We therefore cannot be certain that all participants correctly responded to these variables. Furthermore, the results need to be considered with caution due to the relationship between a possible increase in LD on a specific side and the location of pain on that side. Our study did not properly record these factors nor the participants’ dominant side. To avoid this in future, studies should collect the symptoms’ laterality and the participants’ dominant side as variables of interest.

Another limitation is that the entire sample consisted of women. We recruited only women because the literature indicated that the risk of developing TMDs was 2-fold higher for women than for men (28). The lack of knowledge about the influence of sex and psychological and hormonal factors can be considered a limitation of this study. Lastly, the influence of the nociceptive process on the central nervous system could be another limitation (29).
